# Association of the triglyceride–glucose index with the severity of lower extremity arterial disease across glucose metabolic states: a single-center cross-sectional study of 656 patients with angiographically confirmed disease

**DOI:** 10.3389/fendo.2026.1957246

**Published:** 2026-10-23

**Authors:** Xiaoli Zhou, Dan Zhao, Hong Lei, Gang Zhao, Liping Sha, Xixuan Lu, Haiyan Liang

**Affiliations:** 1 Department of Endocrinology, Cardiovascular and Cerebrovascular Disease Hospital, General Hospital of Ningxia Medical University, Yinchuan, Ningxia, China; 2 Department of Vascular Surgery, General Hospital of Ningxia Medical University, Yinchuan, Ningxia, China

**Keywords:** cross-sectional study, glucose metabolic states, ischemic tissue loss, lower extremity arterial disease, Rutherford classification, triglyceride–glucose index

## Abstract

**Objective:**

To examine the cross-sectional association between the triglyceride–glucose (TyG) index and the severity of lower extremity arterial disease (LEAD) in hospitalized patients with angiographically confirmed LEAD, to test whether this association differs across Glucose Metabolic States, and to quantify the ability of the TyG index to discriminate prevalent ischemic tissue loss (Rutherford grades 5–6).

**Methods:**

This single-center, retrospective, cross-sectional study included 656 consecutive patients with angiographically confirmed LEAD who were hospitalized in the Department of Vascular Surgery at the General Hospital of Ningxia Medical University from January 2023 through December 2024. Diagnosis was confirmed by digital subtraction angiography (DSA). Patients were classified into four groups according to their Glucose Metabolic States: normal glucose tolerance (NGT, n = 150), impaired glucose regulation (IGR, n = 148), diabetes with a duration of <10 years (DM<10y, n = 174), and diabetes with a duration of ≥10 years (DM≥10y, n = 184). Glucose and lipid parameters and LEAD severity (Rutherford classification) were assessed during the same hospitalization. Spearman correlation, multinomial logistic regression (with grade 1 mild claudication as the reference), ordinal logistic regression with a TyG × Glucose Metabolic States interaction term, proportional-odds assessment by cumulative binary splits, ROC analysis with bootstrap internal validation (1,000 resamples), and decision-curve analysis were performed. Sensitivity analyses used continuous glycemic measures instead of the categorical groups, excluded implausibly low fasting glucose values, and added liver enzymes to the adjustment set. The primary outcome was ischemic tissue loss, defined as Rutherford grades 5–6 (n = 205, 31.2%).

**Results:**

The TyG index increased progressively across Glucose Metabolic States categories (8.79 ± 0.51, 9.15 ± 0.48, 9.57 ± 0.56, and 9.67 ± 0.52 for NGT, IGR, DM < 10 years, and DM ≥ 10 years, respectively; P < 0.001) and was strongly correlated with Rutherford grade (Spearman r = 0.677, P < 0.001). In a multinomial logistic regression model adjusted for age, sex, smoking, hypertension, BMI, and fatty liver disease (all variance inflation factors < 1.5), the TyG index remained associated with each severity category above the reference, with adjusted odds ratios per 0.5-unit increment of 2.51 (grade 2), 2.83 (grade 3), 7.56 (grade 4), 9.62 (grade 5), and 20.45 (grade 6). The interaction between TyG and Glucose Metabolic States was statistically significant (likelihood-ratio χ² = 95.63, df = 3, P < 0.001); stratum-specific odds ratios per 0.5-unit increment were 1.51 (95% CI 1.07–2.13) in IGR, 2.50 (1.90–3.29) in DM < 10 years, and 8.14 (5.23–12.67) in DM ≥ 10 years, whereas the NGT-stratum estimate was unstable (quasi-complete separation) and could not be interpreted. When the categorical groups were replaced with a continuous glycemic measure, the interaction with fasting plasma glucose remained significant but was in the opposite direction (χ² = 9.36, df = 1, P = 0.002): the TyG–severity association was stronger at lower fasting glucose levels (OR per 0.5 unit 4.81 at 4.8 mmol/L) than at higher fasting glucose levels (3.46 at 8.3 mmol/L). For discriminating Rutherford grades 5–6, the AUC was 0.833 (95% CI 0.796–0.870); the Youden-optimal cutoff was 9.42 (sensitivity 85.9%, specificity 71.0%, positive predictive value 57.3%, negative predictive value 91.7%, LR + 2.96, LR− 0.199), and this cutoff was stable across bootstrap resamples. Decision-curve analysis showed a positive net benefit relative to treat-all or treat-none strategies across threshold probabilities of approximately 0.09–0.57.

**Conclusion:**

In this cohort of patients with angiographically confirmed LEAD, the TyG index was independently associated with LEAD severity. The strength of this association appeared to vary across Glucose Metabolic States, but the apparent gradient from normal glucose tolerance to long-standing diabetes was not reproduced when glucose was modelled continuously, suggesting that part of the categorical pattern is attributable to the grouping itself (i.e., mathematical coupling and range restriction). The TyG index showed moderate ability to discriminate prevalent ischemic tissue loss. Because exposure and outcome were assessed during the same hospitalization, these findings represent a cross-sectional association rather than a predictive one; the proposed cutoff values were derived from the same sample in which they were evaluated and require external validation before they can be considered for clinical use.

## 1 Introduction

The triglyceride–glucose (TyG) index, calculated from fasting plasma glucose and triglyceride concentrations, is a simple surrogate marker of insulin resistance. Unlike the homeostasis model assessment of insulin resistance (HOMA-IR) and the hyperinsulinemic–euglycemic clamp, the TyG index does not require insulin measurement, is inexpensive, and is highly reproducible, making it suitable for large-scale clinical and epidemiological use. In recent years, the TyG index has been increasingly recognized as a marker of cardiovascular risk (1–3). A recent meta-analysis reported that an elevated TyG index was associated with an increased risk of lower extremity arterial disease (LEAD) (RR = 1.42, 95% confidence interval [CI] 1.21–1.67, P < 0.001), with a stronger association in patients with diabetes (RR = 1.38) than in those without diabetes (RR = 1.06) (4). Throughout this manuscript, disease of the lower limb arteries is referred to as lower extremity arterial disease (LEAD); the term peripheral arterial disease is used only when it appears in the title or terminology of a cited source.

Diabetic foot ulcer (DFU) is among the most common and severe complications of diabetes, affecting approximately 6.3% of people with diabetes worldwide and carrying a lifetime risk of up to 34%. It is a leading cause of disability, hospitalization, and non-traumatic lower-limb amputation (5). LEAD is the principal pathological basis of DFU and contributes to impaired wound healing, gangrene, and limb loss. LEAD is more prevalent in patients with diabetes than in the general population and often remains clinically silent in its early stages while progressing rapidly once established; after ischemic ulceration has developed, treatment options are limited and outcomes are poor (6, 7). Recognition and timely intervention are therefore important. The Chinese Clinical Practice Guidelines for the Prevention and Management of Diabetic Foot (2025 edition) recommend annual assessment for LEAD—including pedal pulse examination and ankle–brachial index (ABI) measurement—in all patients with diabetes aged ≥40 years, and ABI assessment in any patient with clinical signs of limb ischemia and/or foot ulceration (8).

Accumulating evidence supports an association between the TyG index and LEAD severity (9, 10). In a study of 3,176 patients with type 2 diabetes mellitus (T2DM), the prevalence of LEAD differed across quartiles of the TyG index (Q4 > Q3 > Q2 > Q1; P < 0.05), with an area under the receiver operating characteristic curve (AUC) of 0.583 (11). Another study reported an AUC of 0.751 for LEAD in patients with T2DM, which increased to 0.872 when the TyG index was combined with the urinary albumin-to-creatinine ratio (10). The TyG index has also been reported as an independent correlate of DFU recurrence (fully adjusted OR = 3.697, 95% CI 2.099–6.511, P < 0.001), and a TyG index ≥9.65 has been associated with sudden cardiac death in patients with DFU (hazard ratio 75.98, 95% CI 9.16–630.40, P < 0.001) (12, 13).

Several questions remain unresolved (14–16). In particular, whether the association between the TyG index and LEAD severity differs across Glucose Metabolic States categories—including normal glucose tolerance, impaired glucose regulation, and diabetes of different durations—has not been systematically evaluated in a single cohort with angiographically confirmed LEAD (17, 18). Clarifying this issue may inform metabolism-specific risk assessment. In the present study, we therefore examined the cross-sectional association between the TyG index and LEAD severity across four Glucose Metabolic States categories, and we quantified the ability of the TyG index to discriminate prevalent ischemic tissue loss (Rutherford grades 5–6). We emphasize at the outset that both the exposure and the outcome were ascertained during the same hospitalization; the study therefore addresses association and concurrent discrimination, not prediction of incident events.

## 2 Methods

### 2.1 Study design and study population

This was a single-center, retrospective, cross-sectional study. We reviewed consecutive patients hospitalized in the Department of Vascular Surgery at the Cardiovascular and Cerebrovascular Hospital, General Hospital of Ningxia Medical University, between January 2023 and December 2024. A total of 656 patients with angiographically confirmed LEAD, established by digital subtraction angiography (DSA), were included. Because fasting laboratory measurements and the clinical severity assessment were obtained during the same admission, this analysis describes concurrent (cross-sectional) associations; no follow-up data on incident ulceration or limb events were available(**Figure 1**).

**Figure 1:**
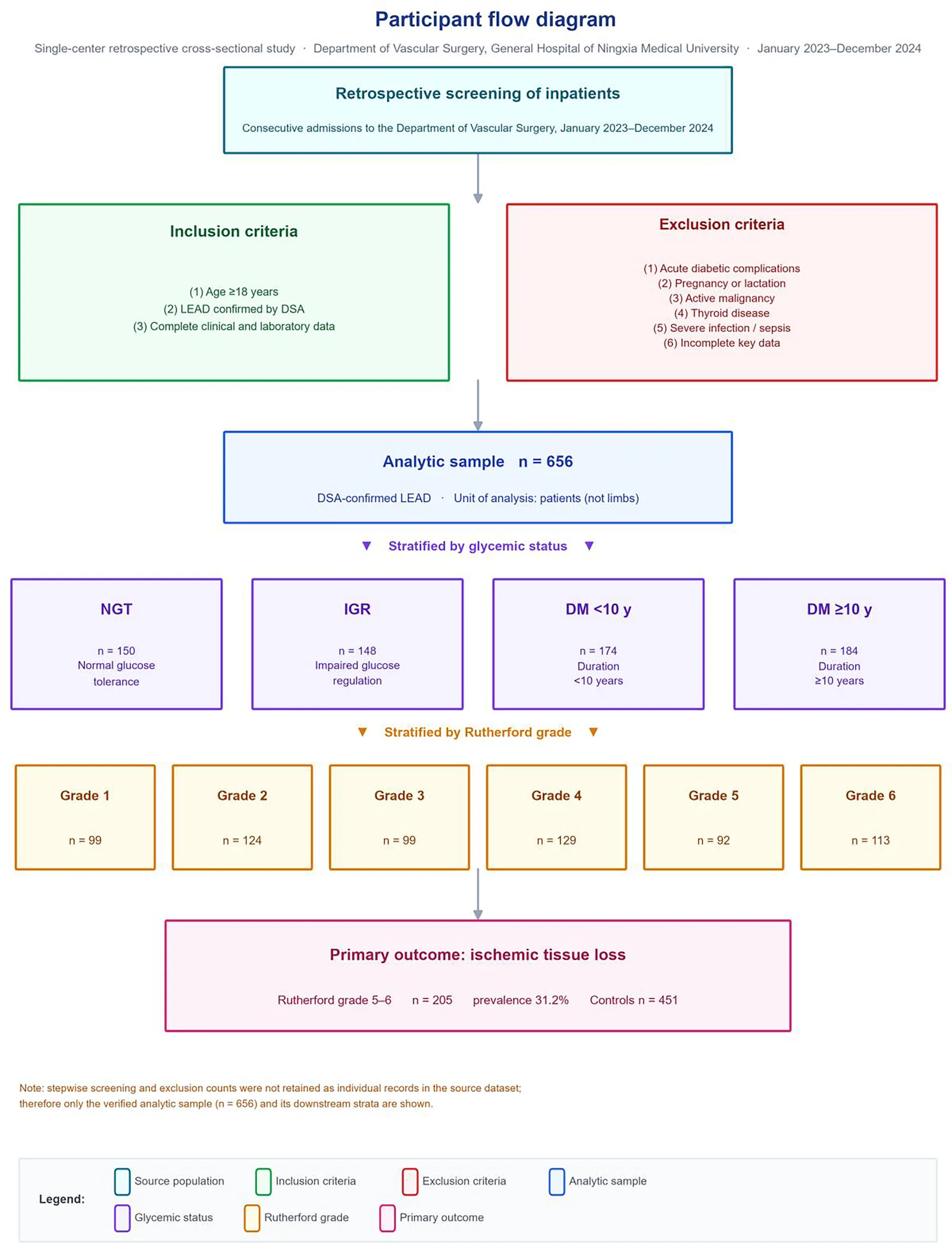
Participant flow diagram. This was a single-center, retrospective, cross-sectional study conducted in the Department of Vascular Surgery, General Hospital of Ningxia Medical University, between January 2023 and December 2024. Consecutive inpatients aged ≥18 years with DSA-confirmed LEAD and complete clinical and laboratory data were included. The final analytic sample comprised 656 patients (unit of analysis: patients, not limbs), stratified by glycemic status: NGT (n = 150), IGR (n = 148), DM <10 y (n = 174), and DM ≥10 y (n = 184). Rutherford grades were distributed as follows: grade 1, n = 99; grade 2, n = 124; grade 3, n = 99; grade 4, n = 129; grade 5, n = 92; and grade 6, n = 113. The primary outcome, ischemic tissue loss (Rutherford grade 5–6), was present in 205 patients (31.2%); 451 patients served as controls. Stepwise screening and exclusion counts were not retained as individual records and are therefore not shown. DSA, digital subtraction angiography; LEAD, lower extremity arterial disease; NGT, normal glucose tolerance; IGR, impaired glucose regulation; DM, diabetes mellitus.

### 2.2 Inclusion and exclusion criteria

Patients were eligible if they met all of the following criteria: (1) age ≥18 years; (2) angiographically confirmed LEAD (DSA); and (3) availability of complete clinical and laboratory data.

Patients were excluded if they had any of the following: (1) acute diabetic complications, including diabetic ketoacidosis or hyperosmolar hyperglycemic state; (2) pregnancy or lactation; (3) active malignancy; (4) clinically diagnosed thyroid disorders; (5) severe systemic infection or sepsis; or (6) incomplete key clinical or laboratory data.

### 2.3 Group assignment and glycemic definitions

Patients were classified into four groups according to Glucose Metabolic States and diabetes duration: (1) normal glucose tolerance (NGT, n = 150); (2) impaired glucose regulation (IGR, n = 148); (3) diabetes with a duration of <10 years (DM<10y, n = 174); and (4) diabetes with a duration of ≥10 years (DM≥10y, n = 184). Classification followed the Guidelines for the Prevention and Treatment of Type 2 Diabetes Mellitus in China (2020 edition): NGT was defined as fasting plasma glucose (FPG) < 6.1 mmol/L without a history of diabetes; IGR as FPG 6.1–6.9 mmol/L without a history of diabetes (impaired fasting glucose) or documented impaired glucose tolerance; and diabetes by a documented physician diagnosis, with duration taken from the medical record. Diabetes duration was abstracted from the medical record at the time of grouping; the continuous duration value was not retained in the analytic dataset, so a formal description of the duration distribution (median, interquartile range) cannot be provided. Diabetes status was based on documented history and the FPG value obtained during the index admission; glycated hemoglobin (HbA1c) and glucose-lowering treatment were not included in the analytic dataset, so the influence of treatment on duration classification could not be examined. Because the TyG index itself contains FPG while the groups are defined partly by glycemic measures, the categorical grouping is structurally dependent on the exposure; we therefore repeated the effect-modification analysis using continuous glycemic measures, as described in Section 2.6.

### 2.4 Clinical data collection and laboratory measurements

Demographic and clinical characteristics included age, sex, height, weight, smoking status, hypertension, and fatty liver disease. After an overnight fast of at least 10 hours, 3 mL of fasting venous blood was collected the following morning. Laboratory measurements included FPG, total cholesterol (TC), triglycerides (TG), low-density lipoprotein cholesterol (LDL-C), high-density lipoprotein cholesterol (HDL-C), uric acid (UA), and the liver enzymes aspartate aminotransferase, alanine aminotransferase, and γ-glutamyl transferase, as well as total bilirubin. Body mass index (BMI) was calculated as weight (kg) divided by height squared (m²). The TyG index was calculated as ln [TG (mg/dL) × FPG (mg/dL)/2]. Information on glucose-lowering treatment (including insulin), lipid-lowering treatment, antiplatelet treatment, renal function, inflammatory markers, HbA1c, neuropathy, and ankle–brachial or toe pressure indices was not available in the analytic dataset and therefore could not be included as covariates.

### 2.5 Assessment of LEAD severity and definition of the outcome

LEAD severity was graded using the Rutherford classification (19, 20): grade 1, mild intermittent claudication (pain-free walking distance >500 m); grade 2, moderate intermittent claudication (300–500 m); grade 3, severe intermittent claudication (<300 m); grade 4, rest pain; grade 5, minor tissue loss or active ulceration; and grade 6, major tissue loss or gangrene. These category definitions follow the published consensus documents, and the term “Rutherford grade” is used consistently throughout; the collective term “severity category” is used only when all six grades are considered together. Severity was abstracted retrospectively from the admission records by two trained abstractors, and disagreements were resolved by a third reviewer (G.Z.). The assessors were not formally blinded to laboratory values; severity assessment and laboratory measurement were both performed during the index admission, and the sequence of the two could not be reconstructed from the dataset. All analyses were conducted at the patient level.

The primary outcome was ischemic tissue loss, defined as Rutherford grades 5–6 (n = 205, 31.2% of the cohort). Ulceration (grade 5) and gangrene (grade 6) were also analyzed separately. A binary “ulcer” indicator in the source dataset was checked against the source records during data verification: 19 records disagreed with the Rutherford-based definition and were realigned, and 4 records (2 patients graded 1–3 with a positive flag) were judged to be flagging errors and were corrected. After verification, the indicator agreed fully with the Rutherford-based definition (205 positive) and was therefore not analyzed as a separate outcome. The Rutherford category alone cannot distinguish among ischemic, neuropathic, neuroischemic, traumatic, and infected ulcers; validated limb-threat or wound classification systems such as WIfI (21), PEDIS, SINBAD, or the University of Texas system could not be applied because ulcer location, depth, duration, infection status, osteomyelitis, and perfusion measurements (toe pressure and transcutaneous oxygen pressure) were not recorded in the analytic dataset.

### 2.6 Statistical analysis

Continuous variables are presented as mean ± standard deviation (SD) and compared across groups using one-way ANOVA, and Kruskal–Wallis tests were used to confirm findings for skewed variables (TG, FPG, and UA). The ordinal Rutherford grade is summarized as median (IQR) and compared using the Kruskal–Wallis test; mean ± SD is not used for this variable because the scale lacks interval properties. Categorical variables are presented as counts and compared using the χ² test; pairwise comparisons were performed using Welch t-tests or χ² tests with Benjamini–Hochberg correction. Spearman rank correlation was used to assess associations between clinical variables and Rutherford grade, with Glucose Metabolic States coded 0–3 (NGT to DM≥10y). Multinomial logistic regression with mild claudication (grade 1) as the reference category was used to estimate the association between the TyG index and each severity category, both unadjusted and adjusted for age, sex, smoking status, hypertension, BMI, and fatty liver disease; a fully extended model that additionally included SBP, DBP, TC, LDL-C, HDL-C, and UA was fitted as a sensitivity analysis (complete estimates for the primary model are provided in **Supplementary Table S1**; the extended model is reported in **Supplementary Table S4**). Because the TyG index is derived from FPG and TG, these two variables were intentionally not included as covariates. Multicollinearity among the covariates in the primary model was assessed using variance inflation factors (VIF). Missing data were negligible (<1% for all analysis variables), and no imputation was performed.

Ordinal logistic regression that included a TyG × Glucose Metabolic States interaction term was used to test for effect modification; the interaction was assessed using a likelihood-ratio test against the model without interaction terms, and stratum-specific estimates were obtained from models fitted within each group. Because the categorical grouping is structurally related to the exposure, three pre-specified sensitivity analyses were performed: (i) the categorical groups were replaced with a continuous glycemic measure (FPG), and the TyG × FPG interaction was tested in an ordinal model; (ii) the same approach was applied using continuous TG; and (iii) the main analyses were repeated after excluding patients with implausibly low FPG values and after adding liver enzymes (aspartate aminotransferase, alanine aminotransferase, γ-glutamyl transferase, and total bilirubin) to the adjustment set. The proportional-odds assumption was examined by comparing cumulative binary logistic models at each split point (grades ≥2, ≥3, ≥4, ≥5, and ≥6). Because ORs expressed per 1-unit increase in the TyG index were numerically large, results are also reported per 0.5-unit increment and per 1 SD (SD = 0.626) of the TyG index. Sparse-data instability was assessed by inspecting the magnitude of the intercepts and the width of the confidence intervals.

Discrimination of prevalent ischemic tissue loss was assessed using ROC analysis. The AUC and its 95% CI were calculated using the Hanley–McNeil method, and internal validation was performed with 1,000 bootstrap resamples to estimate optimism in the AUC and the stability of the Youden-optimal cutoff. Sensitivity, specificity, Youden index, positive and negative predictive values, and positive and negative likelihood ratios were computed using 2 × 2 tables at each candidate cutoff. Decision-curve analysis was used to compare net benefit across threshold probabilities; net benefit was computed for TyG cutoffs of 8.52, 8.91, 9.24, and 9.42 and compared with treat-all and treat-none strategies (**Supplementary Table S3**). Calibration was not assessed because no prediction model was fitted; this analysis quantifies concurrent discrimination only. Statistical analyses were performed using Python 3.13 (statsmodels 0.15.0, SciPy, pandas) and cross-checked in SPSS 22.0 (IBM Corp., Armonk, NY, USA). Two-sided P values < 0.05 were considered statistically significant (**Figure 2**).

**Figure 2:**
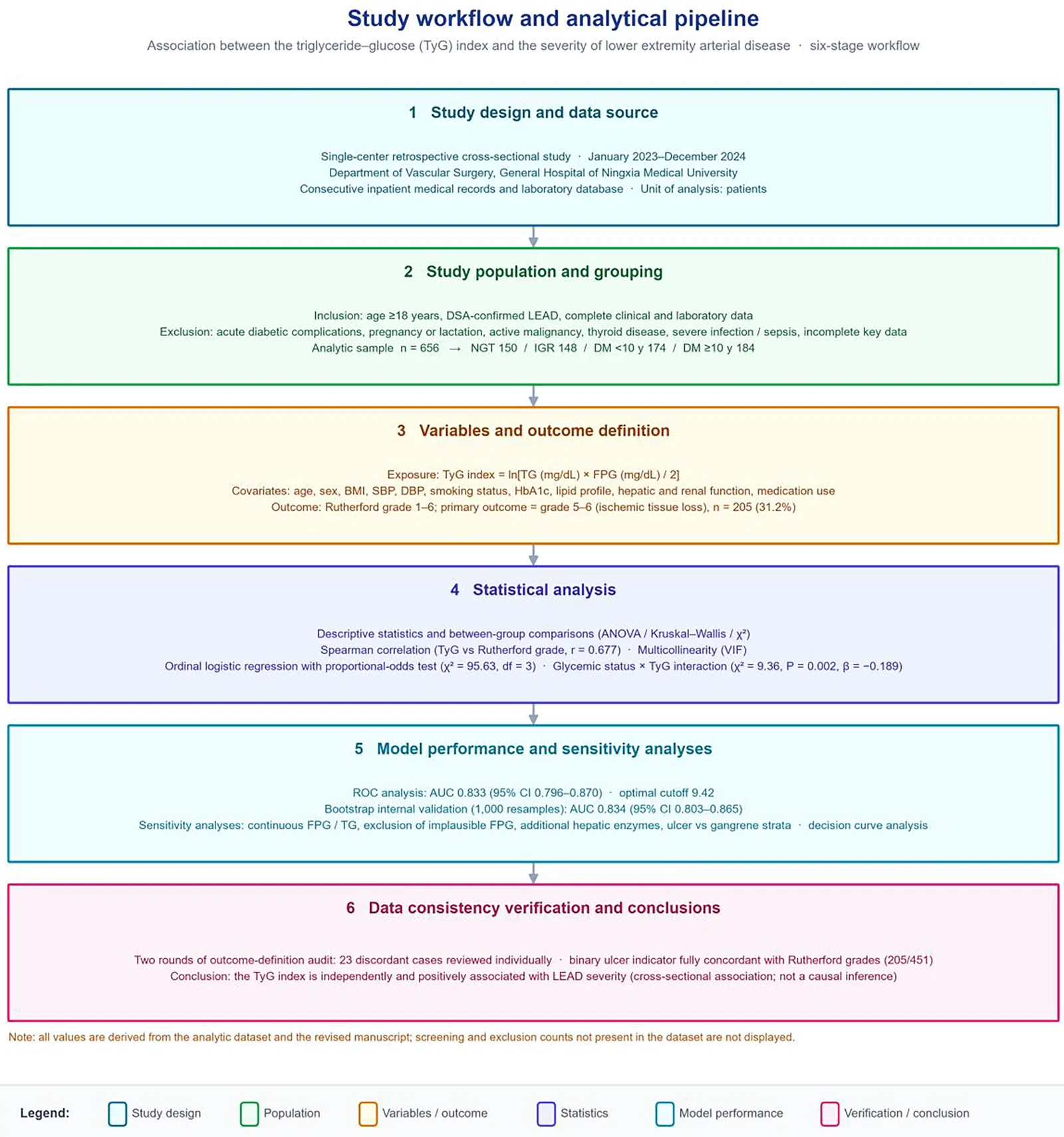
Study workflow and analytical pipeline. The study comprised six sequential stages: (1) study design and data source; (2) study population and grouping; (3) variables and outcome definition; (4) statistical analysis; (5) model performance and sensitivity analyses; and (6) data consistency verification and conclusions. Key methodological details are summarized within each stage. The exposure was the TyG index, calculated as ln[TG (mg/dL) × FPG (mg/dL)/2]. The primary outcome was Rutherford grade 5–6 (ischemic tissue loss; n = 205, 31.2%). ROC analysis yielded an AUC of 0.833 (95% CI 0.796–0.870; optimal cutoff 9.42), and bootstrap internal validation with 1,000 resamples yielded a mean AUC of 0.834 (95% CI 0.803–0.865). AUC, area under the receiver operating characteristic curve; CI, confidence interval; DSA, digital subtraction angiography; FPG, fasting plasma glucose; LEAD, lower extremity arterial disease; NGT, normal glucose tolerance; IGR, impaired glucose regulation; DM, diabetes mellitus; TG, triglycerides; TyG, triglyceride–glucose; VIF, variance inflation factor.

## 3 Results

### 3.1 Baseline characteristics across glucose metabolic states

The baseline characteristics of the 656 patients are shown in **Table 1**. Sex distribution, age, smoking history, and DBP did not differ significantly among the four groups (P > 0.05). Fatty liver disease and hypertension were more common in both diabetes groups than in the NGT and IGR groups, and BMI, TG, TC, LDL-C, and UA differed significantly across groups (P < 0.05). SBP was higher in the DM≥10y group than in the DM<10y group (135.91 ± 18.37 vs 129.94 ± 16.87 mmHg; pairwise P = 0.009). HDL-C was lower in the two diabetes groups than in the NGT group (P = 0.003 and P = 0.005, respectively).

**Table 1:** Baseline clinical characteristics by glucose metabolic states (n = 656).

Characteristic	NGT (n = 150)	IGR (n = 148)	DM<10y (n = 174)	DM≥10y (n = 184)	P value
Sex (male/female)	83/67	75/73	94/80	97/87	0.869
Age (years)	61.75 ± 8.65	60.42 ± 8.43	59.23 ± 8.16	60.06 ± 8.02	0.055
Fatty liver (no/yes)	116/34	102/46	68/106	87/97	<0.001
Hypertension (no/yes)	57/93	42/106	40/134	36/148	0.001
Smoking (no/yes)	74/76	61/87	62/112	77/107	0.100
SBP (mmHg)	133.44 ± 20.63	134.11 ± 17.74	129.94 ± 16.87	135.91 ± 18.37	0.021
DBP (mmHg)	81.77 ± 12.83	80.94 ± 11.00	82.19 ± 10.84	82.61 ± 10.17	0.578
BMI (kg/m²)	24.14 ± 2.72	24.65 ± 2.85	27.24 ± 2.01	27.01 ± 2.93	<0.001
TG (mmol/L)	1.97 ± 1.09	2.20 ± 1.17	2.70 ± 1.39	2.82 ± 1.31	<0.001
TC (mmol/L)	4.08 ± 1.03	4.48 ± 1.13	4.33 ± 1.07	4.68 ± 1.28	<0.001
LDL-C (mmol/L)	2.55 ± 0.79	2.92 ± 0.87	2.80 ± 0.86	2.90 ± 1.08	0.001
HDL-C (mmol/L)	1.00 ± 0.21	0.95 ± 0.24	0.91 ± 0.22	0.92 ± 0.24	0.004
UA (μmol/L)	309.64 ± 79.24	323.38 ± 89.19	346.66 ± 93.79	330.86 ± 82.10	0.002
FPG (mmol/L)	4.78 ± 0.70	6.07 ± 0.90	7.70 ± 2.33	8.32 ± 2.87	<0.001
TyG index	8.79 ± 0.51	9.15 ± 0.48	9.57 ± 0.56	9.67 ± 0.52	<0.001
Rutherford grade	2.0 (1.0–4.0)	2.0 (2.0–3.0)	4.0 (3.0–5.0)	5.0 (4.0–6.0)	<0.001

Values are mean ± SD, median (interquartile range), or counts. Continuous variables were compared using one-way ANOVA and categorical variables using the χ² test; because Rutherford grade is an ordinal scale, that row is summarized as median (interquartile range) and compared using the Kruskal–Wallis test. BMI, body mass index; DBP, diastolic blood pressure; FPG, fasting plasma glucose; HDL-C, high-density lipoprotein cholesterol; IGR, impaired glucose regulation; LDL-C, low-density lipoprotein cholesterol; NGT, normal glucose tolerance; SBP, systolic blood pressure; TC, total cholesterol; TG, triglycerides; TyG, triglyceride–glucose; UA, uric acid.

The TyG index increased progressively across Glucose Metabolic States categories (8.79 ± 0.51 in NGT, 9.15 ± 0.48 in IGR, 9.57 ± 0.56 in DM<10y, and 9.67 ± 0.52 in DM≥10y; P < 0.001); all pairwise comparisons were significant except DM<10y versus DM≥10y (P = 0.067). The distribution of TyG values across the four groups was 8.79 (8.42–9.04), 9.16 (8.85–9.49), 9.64 (9.11–9.94), and 9.75 (9.42–10.00) (median, IQR). Rutherford grade also differed significantly across groups (medians 2.0, 2.0, 4.0, and 5.0; Kruskal–Wallis P < 0.001), with the difference concentrated between the non-diabetic groups and the two diabetes groups.

### 3.2 Correlation analysis

Spearman correlation analysis (**Table 2**) showed that the TyG index was most strongly correlated with Rutherford grade (r = 0.677, P < 0.001), followed by TG (r = 0.553), FPG (r = 0.494), Glucose Metabolic States (r = 0.473), BMI (r = 0.323), and fatty liver disease (r = 0.276). TC (r = 0.204), hypertension (r = 0.136), HDL-C (r = −0.130), UA (r = 0.117), and LDL-C (r = 0.111) were more weakly associated. Age was weakly and inversely correlated with Rutherford grade (r = −0.106, P = 0.007), a finding that may reflect survivor or referral bias in a hospitalized vascular-surgery population and is discussed further below. Sex (r = 0.002, P = 0.961), smoking (r = 0.066, P = 0.093), SBP (r = 0.056, P = 0.153), and DBP (r = 0.070, P = 0.075) were not significantly correlated with LEAD severity.

**Table 2:** Spearman correlations with lower extremity arterial disease (LEAD) severity (Rutherford grade).

Variable	r	P value
TyG index	0.677	<0.001
TG	0.553	<0.001
FPG	0.494	<0.001
Glucose Metabolic States	0.473	<0.001
BMI	0.323	<0.001
Fatty liver disease	0.276	<0.001
TC	0.204	<0.001
Hypertension	0.136	<0.001
HDL-C	−0.130	<0.001
UA	0.117	0.003
LDL-C	0.111	0.004
Age	−0.106	0.007
DBP	0.070	0.075
Smoking	0.066	0.093
SBP	0.056	0.153
Sex	0.002	0.961

“Glucose Metabolic States” is the four-level variable (0 = NGT, 1 = IGR, 2 = DM<10y, 3 = DM≥10y) entered as an ordinal score. BMI, body mass index; DBP, diastolic blood pressure; FPG, fasting plasma glucose; HDL-C, high-density lipoprotein cholesterol; LDL-C, low-density lipoprotein cholesterol; SBP, systolic blood pressure; TC, total cholesterol; TG, triglycerides; TyG, triglyceride–glucose; UA, uric acid.

### 3.3 Multinomial logistic regression

In a multinomial logistic regression model with mild claudication (grade 1, n = 99) as the reference category, the TyG index was associated with each higher severity category (**Table 3**). Adjusted ORs per 1-unit increment were 6.30 (95% CI 3.22–12.32) for moderate claudication, 8.04 (3.92–16.44) for severe claudication, 57.14 (25.54–127.83) for rest pain, 92.58 (38.44–222.98) for ulceration, and 418.04 (163.29–1070.23) for gangrene. Because these estimates were large, we also report them per 0.5-unit increment (2.51, 2.83, 7.56, 9.62, and 20.45, respectively) and per 1 SD (3.16, 3.68, 12.59, 17.02, and 43.74, respectively). The number of patients in each severity category is shown in **Table 3**, and all variance inflation factors in the primary model were below 1.5 (age 1.17, sex 1.38, smoking 1.46, hypertension 1.06, BMI 1.30, fatty liver 1.42, TyG 1.23), indicating no problematic multicollinearity. The model (McFadden pseudo-R² = 0.193) also produced very large intercepts for the advanced categories (−41.3 for rest pain, −47.8 for ulceration, and −59.6 for gangrene), consistent with sparse-data instability along the severity gradient; among the covariates, BMI and fatty liver disease were independently associated with ulceration and gangrene, whereas age, sex, smoking, and hypertension were not (**Supplementary Table S1**). An extended model that additionally adjusted for SBP, DBP, TC, LDL-C, HDL-C, and UA yielded broadly similar estimates for the TyG index (adjusted ORs per 1 unit: 6.80, 9.05, 66.93, 133.47, and 492.42), indicating that the association was not explained by the additional lipid, blood pressure, or urate variables. When implausibly low FPG values were excluded (n = 654) or liver enzymes were added to the adjustment set, the TyG estimates were likewise largely unchanged (per 0.5 unit: 2.61, 2.99, 8.10, 10.90, 21.80 and 2.61, 2.92, 7.83, 10.30, 22.76, respectively). Complete results for the extended model, the collinearity assessment, and all sensitivity analyses are provided in **Supplementary Table S4**.

**Table 3:** Multinomial logistic regression of the TyG index for LEAD severity (reference category: grade 1, mild claudication).

Severity category	Unadjusted OR (95% CI)	Adjusted OR per 1 TyG unit (95% CI)	Adjusted P	Adjusted OR per 0.5 unit	Adjusted OR per 1 SD (0.626)
Moderate claudication (grade 2, n = 124)	7.04 (3.75–13.22)	6.30 (3.22–12.32)	<0.001	2.51	3.16
Severe claudication (grade 3, n = 99)	9.22 (4.71–18.05)	8.04 (3.92–16.44)	<0.001	2.83	3.68
Rest pain (grade 4, n = 129)	67.71 (31.61–145.05)	57.14 (25.54–127.83)	<0.001	7.56	12.59
Ulceration (grade 5, n = 92)	122.53 (53.13–282.60)	92.58 (38.44–222.98)	<0.001	9.62	17.02
Gangrene (grade 6, n = 113)	490.10 (198.58–1209.57)	418.04 (163.29–1070.23)	<0.001	20.45	43.74

Adjusted for age, sex, smoking status, hypertension, BMI, and fatty liver disease (complete estimates in **Supplementary Table S1**). BMI, body mass index; CI, confidence interval; LEAD, lower extremity arterial disease; OR, odds ratio; SD, standard deviation; TyG, triglyceride–glucose.

### 3.4 Association by glucose metabolic states

An ordinal logistic regression model that included a TyG × Glucose Metabolic States interaction term showed statistically significant effect modification (likelihood-ratio χ² = 95.63, df = 3, P < 0.001). Inspection of cumulative binary splits (grade ≥2, ≥3, ≥4, ≥5, ≥6) yielded TyG coefficients of 2.84, 2.45, 3.09, 2.57, and 2.64, of similar magnitude and thus broadly compatible with the proportional-odds assumption.

Stratum-specific estimates are shown in **Table 4**. Per 0.5-unit increment in the TyG index, the OR was 1.51 (95% CI 1.07–2.13) in the IGR group, 2.50 (1.90–3.29) in DM<10y, and 8.14 (5.23–12.67) in DM≥10y. The estimate for the NGT stratum was unstable (OR 99.26, 95% CI 40.60–242.67; coefficient 9.20), consistent with quasi-complete separation; the 95% CI is implausibly wide relative to the other strata and is not interpretable. We therefore report the NGT stratum as uninformative rather than as evidence for the absence or presence of an association, and we flag it for source-data verification.

**Table 4:** Heterogeneity of the TyG–severity association across glucose metabolic states.

Analysis	β	OR per 0.5 TyG unit (95% CI)	P value	Comment
Interaction test (TyG × metabolic status; LR test)	χ² = 95.63 (df = 3)	—	<0.001	Statistically significant heterogeneity across the four groups
NGT stratum (n = 150)	9.196	99.26 (40.60–242.67)	<0.001	Unstable (quasi-complete separation); not interpretable
IGR stratum (n = 148)	0.820	1.51 (1.07–2.13)	0.021	Smallest association
DM<10y stratum (n = 174)	1.833	2.50 (1.90–3.29)	<0.001	—
DM≥10y stratum (n = 184)	4.194	8.14 (5.23–12.67)	<0.001	Largest association among the three interpretable strata
Sensitivity: TyG × continuous FPG (LR test)	χ² = 9.36 (df = 1)	—	0.002	Significant, but negative interaction (β = −0.189): association weaker at higher FPG
Sensitivity: TyG × continuous TG (LR test)	χ² = 14.07 (df = 1)	—	<0.001	Significant interaction with the second TyG component
Proportional-odds check (cumulative splits ≥2/≥3/≥4/≥5/≥6)	2.84/2.45/3.09/2.57/2.64	—	—	Coefficients of similar magnitude; assumption broadly satisfied

CI, confidence interval; DM<10y, diabetes duration <10 years; DM≥10y, diabetes duration ≥10 years; FPG, fasting plasma glucose; IGR, impaired glucose regulation; LR, likelihood ratio; NGT, normal glucose tolerance; OR, odds ratio; SD, standard deviation; TG, triglycerides; TyG, triglyceride–glucose.

β is expressed per 1-unit increment in the TyG index; ORs are expressed per 0.5-unit increment.

Because the categorical grouping is structurally related to the exposure, the interaction was re-examined using continuous glycemic measures in place of the four groups. The TyG × FPG interaction remained statistically significant (likelihood-ratio χ² = 9.36, df = 1, P = 0.002), but the sign of the interaction coefficient was negative (β = −0.189): the TyG–severity association was strongest at low fasting glucose and became progressively weaker as fasting glucose increased (OR per 0.5-unit increment in the TyG index: 4.81 at FPG 4.8 mmol/L, 4.25 at 6.1 mmol/L, 3.66 at 7.7 mmol/L, and 3.46 at 8.3 mmol/L). A TyG × TG interaction was also significant (χ² = 14.07, df = 1, P < 0.001). These results do not reproduce the monotonic increase in effect size suggested by the categorical analysis, and they suggest that the categorical gradient is at least partly attributable to the definition of the groups themselves rather than to a progressively strengthening biological effect. Because continuous FPG does not capture diabetes duration or treatment history, however, a contribution of duration-related biological effects cannot be excluded.

### 3.5 Discrimination of prevalent ischemic tissue loss

For discrimination of Rutherford grades 5–6 (205 of 656 patients; prevalence 31.2%), the TyG index had an AUC of 0.833 (95% CI 0.796–0.870). Bootstrap internal validation yielded a mean AUC of 0.834 (95% CI 0.803–0.865; optimism was negligible), and the Youden-optimal cutoff was 9.42 in the original sample and 9.42 (interquartile range 9.40–9.44) across bootstrap resamples. TyG values were 9.11 ± 0.59 in patients without tissue loss (grades 1–4) and 9.80 ± 0.39 in those with tissue loss (grades 5–6; Mann–Whitney P < 0.001).

**Table 5** summarizes the performance of the three candidate cutoffs proposed in the original submission (8.52, 8.91, and 9.24) together with the Youden-optimal cutoff derived here (9.42). The 8.52 cutoff identified essentially all patients with tissue loss but classified 384 of 451 patients without tissue loss as positive (specificity 14.9%). The 9.42 cutoff provided the best balance (sensitivity 85.9%, specificity 71.0%, positive predictive value 57.3%, negative predictive value 91.7%, LR + 2.96, LR− 0.199). When analyzed separately, ulceration (grade ≥5) and gangrene (grade 6) gave AUCs of 0.833 and 0.834, respectively (**Supplementary Table S2**); after source-data verification, the binary ulcer indicator agreed fully with the Rutherford-based definition (Section 3.6) and was therefore no longer analyzed as a separate outcome.

**Table 5:** Discrimination of prevalent ischemic tissue loss (Rutherford grades 5–6; 205/656, prevalence 31.2%) by candidate TyG cutoffs.

Candidate cutoff	TyG cutoff	Sensitivity (%)	Specificity (%)	Youden index	PPV (%)	NPV (%)	LR+	LR−	TP/FP/FN/TN
High-sensitivity (original submission)	≥8.52	100.0	14.9	0.149	34.8	100.0	1.17	0.000	205/384/0/67
Balanced (original submission)	≥8.91	99.0	36.8	0.358	41.6	98.8	1.57	0.027	203/285/2/166
High-specificity (original submission)	≥9.24	89.8	59.6	0.494	50.3	92.8	2.22	0.172	184/182/21/269
Youden-optimal (this analysis)	≥9.42	85.9	71.0	0.568	57.3	91.7	2.96	0.199	176/131/29/320

AUC 0.833 (95% CI 0.796–0.870); bootstrap-validated AUC 0.834 (95% CI 0.803–0.865). AUC, area under the receiver operating characteristic curve; FN, false negative; FP, false positive; LR+, positive likelihood ratio; LR−, negative likelihood ratio; NPV, negative predictive value; PPV, positive predictive value; TN, true negative; TP, true positive; TyG, triglyceride–glucose.

The intermediate zone between the two thresholds proposed in the original submission (8.52 and 9.24) contained 223 of 656 patients (34.0%), of whom only 21 (9.4%) had ischemic tissue loss; the wider range between the screening threshold and the Youden-optimal cutoff derived here (8.52–9.42) contained 282 patients (43.0%), of whom only 29 (10.3%) had ischemic tissue loss. No management pathway can be defined for either zone based on TyG alone, and because both thresholds are derived from the same single biomarker, neither zone can be resolved by repeating the same measurement.

Decision-curve analysis showed that using TyG ≥9.42 as the threshold for further assessment yielded a higher net benefit than treat-all or treat-none strategies across threshold probabilities of approximately 0.09–0.57 (**Supplementary Table S3**). At very low threshold probabilities (≤0.10), the less specific cutoffs gave marginally higher net benefit, consistent with their higher sensitivity; above approximately 0.15, TyG ≥9.42 was superior. The limited range over which any threshold adds net benefit reflects the fact that the TyG index is a single concurrent laboratory measurement rather than a component of a full diagnostic pathway.

### 3.6 Data consistency checks

Three internal consistency checks were performed. First, the binary ulcer indicator in the source dataset initially disagreed with the Rutherford-based definition of grades 5–6; during source-data verification, each discrepant record was checked against the source records, and the indicator was realigned with the Rutherford-based definition. After verification, the indicator agreed with grades 5–6 for all 656 patients (205 positive), and it was no longer analyzed as a separate outcome. Second, two patients had implausibly low fasting plasma glucose values (1.46 and 2.23 mmol/L); these values were retained in the primary analysis, and a sensitivity analysis excluding them was reported in Section 3.3. Third, all performance metrics reported in this revision were recomputed directly from the verified source dataset; the cutoff-specific sensitivity and specificity values given here supersede those in the original submission, and the analysis code is available from the corresponding author.

## 4 Discussion

### 4.1 Severity-dependent association

The association between the TyG index and LEAD severity was graded across the clinical spectrum: adjusted ORs per 0.5-unit increment increased from 2.51 for moderate claudication to 20.45 for gangrene. This gradient extends previous reports that examined LEAD as a binary outcome rather than by clinical severity category (11, 12, 22). Nevertheless, two features of this gradient warrant cautious interpretation. First, the very large intercepts for the advanced categories indicate that the model is operating near the boundary of the data, so the absolute magnitude of the ORs should not be interpreted as clinically meaningful; effects expressed per 0.5-unit increment or per SD are more interpretable. Second, because TyG is a composite of FPG and TG and the severity gradient parallels Glucose Metabolic States, part of the apparent gradient may reflect the underlying metabolic status itself and mathematical coupling rather than an independent vascular effect.

### 4.2 Glucose metabolic states and the risk of mathematical coupling

Effect modification by Glucose Metabolic States was statistically significant (P < 0.001), with the largest stratum-specific association among the interpretable strata in patients with diabetes with a duration of ≥10 years (OR per 0.5-unit increment: 2.50 in DM<10y vs 8.14 in DM≥10y). This pattern is biologically plausible: prolonged hyperglycemia and insulin resistance may act synergistically on endothelial function, oxidative stress, and plaque progression, and insulin resistance is an established determinant of atherosclerosis through adipose-tissue signaling, inflammatory activation, and advanced glycation end-product formation (23–26) (27–30). A similar modification of the TyG–cardiovascular association by diabetes status has been reported in meta-analyses of coronary heart disease cohorts (31).

However, the TyG index is calculated from fasting plasma glucose, and the groups are defined by glycemic status, so a degree of mathematical coupling is unavoidable. Two observations in this dataset suggest that at least part of this heterogeneity may be artefactual. First, the NGT stratum estimate was unstable and extreme, which suggests that stratum-specific estimates within this cohort are highly sensitive to the underlying data structure. Second, when the categorical grouping was replaced with a continuous glycemic measure, the interaction remained significant but was in the opposite direction: the TyG–severity association was strongest at the lowest fasting glucose values (OR per 0.5-unit increment 4.81 at 4.8 mmol/L) and attenuated as fasting glucose rose (3.46 at 8.3 mmol/L). This discrepancy argues against interpreting the categorical gradient as a purely progressive biological effect of current glycemia, but it does not exclude a contribution of duration-related biological effects, because continuous fasting glucose does not capture diabetes duration or treatment history. We therefore interpret the categorical interaction as evidence of heterogeneity rather than as proof of a progressively increasing biological effect, and we do not present the four-group gradient as a biological conclusion. The concentration of the TyG–severity association within a restricted range of values in each group is also consistent with range restriction, as the TyG distribution differed markedly across groups (median 8.79 in NGT vs 9.75 in DM≥10y). Reporting the association per 0.5-unit increment and per SD mitigates, but does not eliminate, this problem.

### 4.3 Discrimination of prevalent ischemic tissue loss

The TyG index discriminated prevalent tissue loss with an AUC of 0.833, and the Youden-optimal cutoff (9.42) remained stable across bootstrap resampling. The candidate cutoffs proposed in the original submission performed differently from the originally reported values: a cutoff of 8.52 identified all patients with tissue loss but had a specificity of only 14.9%, so that 85% of patients without tissue loss would be referred for further assessment. A cutoff of 9.42 offered a more balanced trade-off, but its positive predictive value was 57.3%, meaning that nearly half of the patients it selected did not have tissue loss. The intermediate zone between the two originally proposed thresholds (8.52 and 9.24) contained 223 patients (34.0%), of whom only 21 (9.4%) had ischemic tissue loss; because both thresholds are derived from the same biomarker, this zone could not be resolved by repeating the same measurement, and no management pathway was proposed for it. Decision-curve analysis showed a positive net benefit over a limited range of threshold probabilities, supporting a possible role as an adjunctive triage variable rather than as a stand-alone screening or confirmatory test. In particular, a sequential “screening then confirmatory” strategy based on two cutoffs of a single index is not statistically defensible, because the two cutoffs are not independent tests.

### 4.4 Relation to current guidance on LEAD and diabetic foot disease

Current international guidance frames advanced LEAD within the broader concept of limb threat. The Global Vascular Guidelines define chronic limb-threatening ischemia by objective hemodynamic criteria combined with wound and infection assessment, and recommend that limb staging use the WIfI system rather than Rutherford category alone (19, 31). The IWGDF guidance likewise structures the assessment of diabetes-related foot disease around combined vascular, neurological, and infection evaluation (21), and the earlier TASC II consensus remains the reference for the clinical classification of LEAD (20). Three implications follow for the present study.

Two features of the Rutherford system constrain the interpretation of our outcome. Grades 5–6 denote limbs with tissue loss; however, they do not confirm that the tissue loss is ischemic, as neuropathic and infected ulcers are common in diabetes and frequently coexist with ischemia (i.e., neuroischemic ulcers) (18, 21). The outcome analyzed here is therefore best described as prevalent tissue loss graded by Rutherford category, and not as angiographically confirmed ischemic ulceration.

A second question is whether TyG provides incremental value beyond routinely performed tests. ABI measurement, pulse examination, duplex ultrasonography, and perfusion assessment remain the standard tools for identifying LEAD, and in patients with diabetes, ABI is recommended as part of clinical assessment rather than as a population screening tool (32, 33). In this cohort, the TyG index was not compared head-to-head with ABI, toe pressure, or imaging findings, because those variables were not recorded; the incremental value of TyG over standard vascular assessment therefore remains untested, and the present analysis cannot establish that TyG adds information beyond routine evaluation.

The risk-factor profile of this cohort is consistent with what is already known about LEAD. Diabetes, obesity, dyslipidemia, and hypertension were all common, as would be expected given their established contributions to both LEAD and amputation risk (24, 34, 35). The TyG index should be understood as one element of metabolic risk assessment in this setting rather than as a substitute for vascular evaluation.

### 4.5 Comparison with previous studies

The TyG index has been examined previously in relation to chronic limb-threatening ischemia and long-term mortality in patients with LEAD (9, 10), and previous studies have reported AUCs of 0.583 and 0.751 in patients with T2DM (11), whereas an AUC of 0.68 has been reported for delayed wound healing in Rutherford grade 5 ischemia (12). The discrimination observed here (AUC 0.833) is higher, most likely because the outcome was prevalent tissue loss in a selected hospitalized population with an already high burden of disease, rather than incident LEAD in a broader diabetes population. Population-specific thresholds have also been reported: a TyG cutoff of 9.72 for coronary heart disease in a Chinese cohort versus 8.51 in a British cohort (33), and a TyG index that predicts cardiovascular risk at different absolute levels in older US and Japanese populations (36, 37). These observations reinforce the need for population-specific and outcome-specific thresholds rather than a single universal value.

### 4.6 Study limitations

Several limitations must be emphasized. (1) Design: this is a retrospective, single-center, cross-sectional analysis in which exposure and outcome were measured during the same hospitalization. No temporal sequence can be established, and associations cannot be interpreted as prediction; reverse causation is plausible, because active ulceration, gangrene, infection, physiological stress, altered diet, and recent treatment can all alter fasting glucose and triglycerides. (2) Spectrum and selection bias: all participants were hospitalized vascular-surgery patients with angiographically confirmed LEAD, a group that is not representative of primary care, community assessment, asymptomatic diabetes, or early-stage LEAD. (3) Unmeasured confounding. Several variables that would materially affect the estimates were missing from the dataset, including HbA1c, glucose-lowering therapy (including insulin), lipid-lowering therapy and antiplatelet treatment, renal function, inflammatory markers, neuropathy, ankle–brachial index and toe pressure, and previous revascularization. We were unable to adjust for any of these variables. A continuous measure of diabetes duration was also not retained, which meant we could not examine how glucose-lowering treatment influenced the classification based on diabetes duration. (4) Outcome definition: Rutherford grade alone cannot distinguish ischemic, neuropathic, neuroischemic, traumatic, and infected ulcers; validated limb-threat classifications (WIfI, PEDIS, SINBAD, University of Texas) could not be applied because ulcer location, depth, duration, infection, osteomyelitis, and perfusion measurements were not recorded, and unit-of-analysis information (patient versus limb, handling of bilateral disease) was not captured. (5) Mathematical coupling and range restriction: because TyG incorporates FPG and the groups are defined by glycemic status, the categorical interaction is structurally dependent on the exposure; the continuous-measure sensitivity analysis suggests that the apparent four-group gradient may not represent a purely progressively strengthening effect, and readers should not interpret the stratum-specific ORs as biological effect sizes. (6) Statistical instability: the TyG–severity association operates near the boundary of the data for advanced categories, with very large intercepts and stratum-specific estimates that become unstable in the NGT group; the analytic dataset also contains two implausibly low fasting glucose values that were retained in the primary analysis, with a sensitivity analysis excluding them reported in Section 3.3. (7) Validation: the proposed cutoffs were derived and evaluated in the same sample. Bootstrap resampling addresses optimism in the AUC but does not replace independent external validation, which is required before any clinical use; calibration was not assessed because no prediction model was fitted. (8) Finally, severity was abstracted retrospectively by assessors who were not blinded to laboratory values, and assessment of the sequence between laboratory sampling and severity grading was not possible from the available records.

### 4.7 Clinical implications

The TyG index is calculated from two routine fasting measurements and is therefore inexpensive and widely available. In the population studied here—hospitalized patients with angiographically confirmed LEAD—a TyG index above approximately 9.4 was associated with a substantially higher concurrent probability of ischemic tissue loss and may serve as one element of risk assessment, particularly in settings where vascular imaging capacity is limited. It should not, however, be used to replace imaging-based or hemodynamic assessment, to screen general or primary-care populations, or to guide care on the basis of the thresholds reported here until they have been validated prospectively in an independent, multicenter sample with a clearly defined temporal sequence between measurement and outcome. We also note that the TyG index is a marker of metabolic risk. Our data do not show that TyG-guided care prevents ulceration, reduces the need for angiography, improves resource allocation, or is cost-effective. These are questions for prospective study.

### 4.8 Conclusions

In patients with angiographically confirmed LEAD, the TyG index was independently associated with disease severity after adjustment for available covariates. Although the strength of this association appeared to differ across categories of Glucose Metabolic States, the apparent gradient from normal glucose tolerance to long-standing diabetes was not reproduced when glycemia was modelled continuously, suggesting that at least part of the categorical pattern may be attributable to the grouping itself. The TyG index discriminated prevalent tissue loss with moderate accuracy (AUC 0.833; Youden-optimal cutoff 9.42). Because the study is cross-sectional and the thresholds were derived without external validation, these findings should be regarded as hypothesis-generating: they support prospective, multicenter evaluation of whether TyG-based risk assessment, embedded in a defined clinical pathway that also includes standard vascular assessment, can improve limb-related outcomes.

## Data Availability

The raw data supporting the conclusions of this article will be made available by the authors, without undue reservation.

## Supplementary material

Supplementary Table 1 Complete estimates of the adjusted multinomial logistic regression model. Reference category: grade 1 (mild claudication). Intercepts are omitted for clarity. An extended model additionally adjusting for SBP, DBP, TC, LDL-C, HDL-C, and UA is available from the authors. BMI, body mass index; CI, confidence interval; OR, odds ratio; TyG, triglyceride–glucose.

Supplementary Table 2 Distribution of the TyG index by Rutherford grade and separate discrimination of ulceration and gangrene. AUC, area under the receiver operating characteristic curve; SD, standard deviation; TyG, triglyceride–glucose.

Supplementary Table 3 Decision-curve analysis: net benefit at different threshold probabilities (outcome: Rutherford grade 5–6; prevalence 31.2%). The net benefit of the treat-none strategy is zero at every threshold and is therefore not tabulated separately. A positive value indicates that the strategy performs better than treating no patient; a value above the treat-all column indicates better performance than treating every patient. TyG, triglyceride–glucose.

Supplementary Table 4 Collinearity assessment and sensitivity analyses. Part 1. Variance inflation factors (VIF) for covariates in the primary model. All VIFs were below 1.5, indicating no collinearity requiring correction. Reference correlations: TyG vs TG, Spearman r = 0.844; TyG vs FPG, r = 0.621; FPG vs TG, r = 0.154. BMI, body mass index; FPG, fasting plasma glucose; TG, triglycerides; TyG, triglyceride–glucose; VIF, variance inflation factor. Part 2. Interaction tests using continuous glycemic indices instead of categorical Glucose Metabolic States (ordinal logistic regression). The interaction was significant but in the direction opposite to the categorical analysis, suggesting that the categorical gradient mainly reflects group definition rather than a graded biological effect. FPG in mmol/L. OR, odds ratio; TyG, triglyceride–glucose. Part 3. Sensitivity analyses excluding implausible values and adding further covariates (multinomial logistic regression; adjusted OR per 0.5 TyG unit). Estimates remained essentially unchanged across all sensitivity analyses; the association between the TyG index and LEAD severity was not explained by lipids, blood pressure, uric acid, or liver enzymes. ALT, alanine aminotransferase; AST, aspartate aminotransferase; DBP, diastolic blood pressure; GGT, γ-glutamyl transferase; HDL-C, high-density lipoprotein cholesterol; LDL-C, low-density lipoprotein cholesterol; SBP, systolic blood pressure; TBIL, total bilirubin; TC, total cholesterol; UA, uric acid. Part 4. Goodness of fit of the TyG index versus its components (ordinal logistic regression; McFadden pseudo-R²). TyG fitted better than either component alone, but it remains highly collinear with TG (r = 0.844), so mathematical coupling cannot be excluded. AIC, Akaike information criterion; HDL-C, high-density lipoprotein cholesterol. Part 5. Intermediate zone and unstable cases for the primary outcome. The TyG cutoffs 8.52, 8.91, 9.24 and 9.42 are expressed in the same units as the TyG index. The 8.52–9.42 interval is the intermediate zone where the primary outcome classification is most sensitive to the cutoff chosen.
