# Supplementary material for: Association of the triglyceride–glucose index with the severity of lower extremity arterial disease across glucose metabolic states: a single-center cross-sectional study of 656 patients with angiographically confirmed disease
Source: Front Endocrinol (Lausanne). 2026 Oct 23;17:1957246. doi: 10.3389/fendo.2026.1957246 (PMC13739561; doi:10.3389/fendo.2026.1957246)
Supplement: Supplementary file 1 [file SupplementaryFile1.docx]

**Supplementary Table S1. Complete estimates of the adjusted multinomial logistic regression model**

| **Outcome category** | **Variable** | **β** | **OR (95% CI)** | **P value** |
| --- | --- | --- | --- | --- |
| Grade 2 | TyG index | 1.840 | 6.296 (3.218–12.316) | <0.001 |
| Grade 2 | Age (per year) | −0.012 | 0.988 (0.953–1.025) | 0.529 |
| Grade 2 | Sex (female vs male) | 0.289 | 1.335 (0.676–2.636) | 0.405 |
| Grade 2 | Smoking (yes vs no) | −0.399 | 0.671 (0.338–1.332) | 0.254 |
| Grade 2 | Hypertension (yes vs no) | 0.304 | 1.355 (0.738–2.487) | 0.327 |
| Grade 2 | BMI (per kg/m²) | 0.024 | 1.025 (0.919–1.142) | 0.661 |
| Grade 2 | Fatty liver (yes vs no) | 0.234 | 1.263 (0.589–2.709) | 0.548 |
| Grade 3 | TyG index | 2.082 | 8.019 (3.912–16.438) | <0.001 |
| Grade 3 | Age (per year) | −0.017 | 0.984 (0.946–1.023) | 0.409 |
| Grade 3 | Sex (female vs male) | 0.378 | 1.460 (0.710–3.001) | 0.304 |
| Grade 3 | Smoking (yes vs no) | −0.663 | 0.515 (0.248–1.070) | 0.075 |
| Grade 3 | Hypertension (yes vs no) | 0.322 | 1.380 (0.714–2.666) | 0.338 |
| Grade 3 | BMI (per kg/m²) | 0.105 | 1.110 (0.989–1.247) | 0.077 |
| Grade 3 | Fatty liver (yes vs no) | 0.068 | 1.071 (0.479–2.395) | 0.868 |
| Grade 4 | TyG index | 4.046 | 57.142 (25.543–127.835) | <0.001 |
| Grade 4 | Age (per year) | 0.018 | 1.019 (0.977–1.062) | 0.385 |
| Grade 4 | Sex (female vs male) | −0.185 | 0.831 (0.391–1.770) | 0.632 |
| Grade 4 | Smoking (yes vs no) | −0.644 | 0.525 (0.242–1.140) | 0.103 |
| Grade 4 | Hypertension (yes vs no) | 0.363 | 1.438 (0.704–2.940) | 0.319 |
| Grade 4 | BMI (per kg/m²) | 0.149 | 1.161 (1.026–1.314) | 0.018 |
| Grade 4 | Fatty liver (yes vs no) | 0.450 | 1.568 (0.697–3.529) | 0.277 |
| Grade 5 | TyG index | 4.528 | 92.577 (38.436–222.980) | <0.001 |
| Grade 5 | Age (per year) | 0.026 | 1.027 (0.980–1.075) | 0.264 |
| Grade 5 | Sex (female vs male) | 0.255 | 1.290 (0.570–2.923) | 0.541 |
| Grade 5 | Smoking (yes vs no) | −0.518 | 0.596 (0.255–1.390) | 0.231 |
| Grade 5 | Hypertension (yes vs no) | 0.016 | 1.016 (0.463–2.227) | 0.968 |
| Grade 5 | BMI (per kg/m²) | 0.177 | 1.193 (1.042–1.367) | 0.011 |
| Grade 5 | Fatty liver (yes vs no) | 0.962 | 2.617 (1.092–6.271) | 0.031 |
| Grade 6 | TyG index | 6.036 | 418.037 (163.287–1070.231) | <0.001 |
| Grade 6 | Age (per year) | 0.011 | 1.011 (0.965–1.059) | 0.644 |
| Grade 6 | Sex (female vs male) | −0.355 | 0.701 (0.301–1.633) | 0.410 |
| Grade 6 | Smoking (yes vs no) | −0.773 | 0.462 (0.192–1.112) | 0.085 |
| Grade 6 | Hypertension (yes vs no) | 0.218 | 1.243 (0.552–2.799) | 0.599 |
| Grade 6 | BMI (per kg/m²) | 0.133 | 1.143 (0.995–1.312) | 0.059 |
| Grade 6 | Fatty liver (yes vs no) | 0.319 | 1.375 (0.570–3.317) | 0.478 |

Reference category: grade 1 (mild claudication). Intercepts are omitted for clarity. An extended model additionally adjusting for SBP, DBP, TC, LDL-C, HDL-C, and UA is available from the authors. BMI, body mass index; CI, confidence interval; OR, odds ratio; TyG, triglyceride–glucose.

**Supplementary Table S2. Distribution of the TyG index by Rutherford grade and separate discrimination of ulceration and gangrene**

| **Analysis** | **n** | **TyG (mean ± SD) or metric** | **Statistic** | **P value** |
| --- | --- | --- | --- | --- |
| Grade 1 (mild claudication) | 99 | 8.60 ± 0.66 | — | — |
| Grade 2 (moderate claudication) | 124 | 9.05 ± 0.44 | — | — |
| Grade 3 (severe claudication) | 99 | 9.11 ± 0.41 | — | — |
| Grade 4 (rest pain) | 129 | 9.55 ± 0.45 | — | — |
| Grade 5 (ulceration) | 92 | 9.66 ± 0.30 | — | — |
| Grade 6 (gangrene) | 113 | 9.91 ± 0.42 | — | — |
| TyG across grades (Kruskal–Wallis) | 656 | — | H = 307.9 | <0.001 |
| Discrimination of ulceration (grade ≥5) | 205 positive / 451 negative | AUC 0.833 | — | <0.001 |
| Discrimination of gangrene (grade 6) | 113 positive / 543 negative | AUC 0.834 | — | <0.001 |
| Binary ulcer indicator after source-data verification | 205 positive / 451 negative | fully concordant with grades 5–6 | — | — |

AUC, area under the receiver operating characteristic curve; SD, standard deviation; TyG, triglyceride–glucose.

**Supplementary Table S3. Decision-curve analysis: net benefit at different threshold probabilities (outcome: Rutherford grade 5–6; prevalence 31.2%)**

| **Threshold probability (pt)** | **Treat all** | **TyG ≥8.52** | **TyG ≥8.91** | **TyG ≥9.24** | **TyG ≥9.42** |
| --- | --- | --- | --- | --- | --- |
| 0.10 | 0.2361 | 0.2475 | 0.2612 | 0.2497 | 0.2461 |
| 0.15 | 0.1912 | 0.2092 | 0.2328 | 0.2315 | 0.2331 |
| 0.20 | 0.1406 | 0.1662 | 0.2008 | 0.2111 | 0.2184 |
| 0.25 | 0.0833 | 0.1174 | 0.1646 | 0.1880 | 0.2017 |
| 0.30 | 0.0179 | 0.0616 | 0.1233 | 0.1616 | 0.1827 |
| 0.35 | −0.0577 | −0.0027 | 0.0755 | 0.1311 | 0.1608 |
| 0.40 | −0.1458 | −0.0777 | 0.0198 | 0.0955 | 0.1352 |
| 0.45 | −0.2500 | −0.1664 | −0.0460 | 0.0535 | 0.1049 |
| 0.50 | −0.3750 | −0.2729 | −0.1250 | 0.0030 | 0.0686 |
| 0.55 | −0.5278 | −0.4029 | −0.2215 | −0.0586 | 0.0242 |
| 0.60 | −0.7187 | −0.5655 | −0.3422 | −0.1357 | −0.0311 |

The net benefit of the treat-none strategy is zero at every threshold and is therefore not tabulated separately. A positive value indicates that the strategy performs better than treating no patient; a value above the treat-all column indicates better performance than treating every patient. TyG, triglyceride–glucose.

**Supplementary Table S4. Collinearity assessment and sensitivity analyses**

**Part 1. Variance inflation factors (VIF) for covariates in the primary model.**

| **Variable** | **VIF** | **Interpretation** |
| --- | --- | --- |
| Age | 1.17 | No collinearity |
| Sex | 1.38 | No collinearity |
| Smoking | 1.46 | No collinearity |
| Hypertension | 1.06 | No collinearity |
| BMI | 1.30 | No collinearity |
| Fatty liver disease | 1.42 | No collinearity |
| TyG index | 1.23 | No collinearity |

All VIFs were below 1.5, indicating no collinearity requiring correction. Reference correlations: TyG vs TG, Spearman r = 0.844; TyG vs FPG, r = 0.621; FPG vs TG, r = 0.154. BMI, body mass index; FPG, fasting plasma glucose; TG, triglycerides; TyG, triglyceride–glucose; VIF, variance inflation factor.

**Part 2. Interaction tests using continuous glycemic indices instead of categorical Glucose Metabolic States (ordinal logistic regression).**

| **Model** | **Test statistic** | **P value** | **TyG slope / OR per 0.5 unit** |
| --- | --- | --- | --- |
| TyG × continuous FPG | χ² = 9.36 (df = 1) | 0.002 | FPG 4.8 → 4.81; 6.1 → 4.25; 7.7 → 3.66; 8.3 → 3.46 (interaction β = −0.189) |
| TyG × continuous TG | χ² = 14.07 (df = 1) | <0.001 | Interaction also present for the other component of the TyG index |
| Interpretation | Interaction significant, but in the direction opposite to the categorical analysis | — | Supports that the categorical gradient arises mainly from the group definition rather than from a graded biological effect |

The interaction was significant but in the direction opposite to the categorical analysis, suggesting that the categorical gradient mainly reflects group definition rather than a graded biological effect. FPG in mmol/L. OR, odds ratio; TyG, triglyceride–glucose.

**Part 3. Sensitivity analyses excluding implausible values and adding further covariates (multinomial logistic regression; adjusted OR per 0.5 TyG unit).**

| **Model** | **Grade 2** | **Grade 3** | **Grade 4** | **Grade 5** | **Grade 6** |
| --- | --- | --- | --- | --- | --- |
| Primary model (n = 656; adjusted for age, sex, smoking, hypertension, BMI, fatty liver) | 2.51 | 2.83 | 7.56 | 9.62 | 20.45 |
| Excluding 2 implausible FPG values (n = 654) | 2.61 | 2.99 | 8.10 | 10.90 | 21.80 |
| Additionally adjusted for AST, ALT, GGT and TBIL | 2.61 | 2.93 | 7.83 | 10.30 | 22.76 |
| Extended model (also SBP, DBP, TC, LDL-C, HDL-C and UA; OR per 1 unit) | 6.80 | 9.05 | 66.93 | 133.47 | 492.42 |

Estimates remained essentially unchanged across all sensitivity analyses; the association between the TyG index and LEAD severity was not explained by lipids, blood pressure, uric acid, or liver enzymes. ALT, alanine aminotransferase; AST, aspartate aminotransferase; DBP, diastolic blood pressure; GGT, γ-glutamyl transferase; HDL-C, high-density lipoprotein cholesterol; LDL-C, low-density lipoprotein cholesterol; SBP, systolic blood pressure; TBIL, total bilirubin; TC, total cholesterol; UA, uric acid.

**Part 4. Goodness of fit of the TyG index versus its components (ordinal logistic regression; McFadden pseudo-R²).**

| **Index** | **pseudo-R²** | **AIC** | **Comment** |
| --- | --- | --- | --- |
| TyG index alone | 0.1835 | 1923.1 | Better fit than the sum of its two components |
| TG + FPG | 0.1485 | 2007.0 | — |
| TG alone | 0.0757 | — | — |
| FPG alone | 0.0607 | — | — |
| HDL-C alone (comparator) | 0.0037 | — | — |

TyG fitted better than either component alone, but it remains highly collinear with TG (r = 0.844), so mathematical coupling cannot be excluded. AIC, Akaike information criterion; HDL-C, high-density lipoprotein cholesterol.

**Part 5. Intermediate zone and unstable cases for the primary outcome.**

| **Item** | **Value** | **Comment** |
| --- | --- | --- |
| 8.52 ≤ TyG < 9.42 | 282 (43.0%) | Only 29 (10.3%) had Rutherford grade 5–6 |
| TyG ≥ 9.42 | 307 | 176 had Rutherford grade 5–6 |
| FPG < 3.0 mmol/L | 2 (1.46 and 2.23 mmol/L) | Excluded in the sensitivity analysis in Part 3 |
| Ulcer indicator discordant with Rutherford grades 5–6 | 0 | After source-data verification the ulcer indicator was fully concordant with Rutherford grades 5–6 (205 positive / 451 negative) |

The TyG cutoffs 8.52, 8.91, 9.24 and 9.42 are expressed in the same units as the TyG index. The 8.52–9.42 interval is the intermediate zone where the primary outcome classification is most sensitive to the cutoff chosen.
